# Integrativesubtractive proteomics, immunoinformatics, docking, and simulation approaches reveal candidate vaccine against Sin Nombre orthohantavirus

**DOI:** 10.3389/fimmu.2022.1022159

**Published:** 2022-11-11

**Authors:** Alhumaidi B. Alabbas

**Affiliations:** Department of Pharmaceutical Chemistry, College of Pharmacy, Prince Sattam Bin Abdulaziz University, Al Kharj, Saudi Arabia

**Keywords:** hantaviruses, Sin Nombre orthohantavirus, vaccine, computational vaccinology, bioinformatics

## Abstract

The emergence of Sin Nombre orthohantavirus, an etiological agent of hantavirus cardiopulmonary syndrome, exacerbates the situation and imposes a heavy financial burden on healthcare organizations. Multidrug-resistant forms of the disease are prevalent, and there is currently no licensed commercial vaccine. Due to the numerous limitations of experimental vaccines, vaccines against various bacterial and viral diseases have developed *via* computational vaccine design. Several subtractive proteomics, immunoinformatics, docking, and simulation approaches were used in this study to develop a multi-epitope–based vaccine against Sin Nombre orthohantavirus. One possible antigenic protein—the glycoprotein precursor of surface glycoproteins (accession number >AAC42202.1)—was selected as a candidate for B cell–derived T cell epitopes mapping the detailed analysis of the core genome. Among the predicted epitopes, four epitopes (QVDWTKKSST, GLAASPPHL, SSYSYRRKLV, and MESGWSDTA), which were probably antigenic, nonallergenic, nontoxic, and water soluble, were used in the multi-epitope vaccine’s construction. The shortlisted epitopes have the potency to cover 99.78% of the world’s population, 97.93% of the Chinese population, and 97.36% of the Indian population. The epitopes were connected through AAY linkers and joined with >50S ribosomal adjuvant to enhance their efficacy. The vaccine comprises 182 amino acids with a molecular weight of 19.03770 kDa and an instability index of 26.52, indicating that the protein is stable. A molecular docking study revealed that the vaccine has a good binding affinity with TLR-4 and TLR-8, which is vital for inducing the immune system. Top-1 docked complexes of vaccine- TLR-4 and TLR-8 with the lowest binding energy of -12.52 kc/mol and -5.42 kc/mol, respectively, were considered for molecular dynamic simulation analysis. Furthermore, we predicted that the docked complexes are properly stable throughout simulation time in both normal mode and AMBER-based simulation analysis. The MMGBSA analysis calculated -122.17 and -125.4 net binding energies for the TLR-8- and TLR4-vaccine complexes, respectively, while the MMPBSA analysis estimated -115.63 and -118.19 for the TLR-8- and TLR4-vaccine complex, respectively, confirming that the binding stability with receptors is stable, which is important for inducing a strong response. However, the current work is computation-based, so experimental validation is highly recommended.

## Introduction

Sin Nombre virus (SNV) is a negative sense RNA virus classified as a class (A) pathogenic agent by the CDC in an updated categorized list of highly pathogenic viruses. It causes hantavirus cardiopulmonary syndrome (HCPS), frequently reported in North America, which has an ∼36% mortality rate (1). SNV is an enveloped virus containing three segmented RNA encoding enzyme RNA-dependent RNA polymerase, glycoprotein precursor, and nucleocapsid protein. Glycoprotein and nucleocapsid play key roles in providing protective immunity (2). SNV was first discovered in 1993 in hanta outbreaks. Individuals who are exposed to Peromyscus maniculatus (American deer mice), the major reservoir of SNV, have a greater chance of infection. SNV has the aerosol route of transmission and is highly lethal, which makes it a good bioterrorism agent (3).

HCPS is a rare but serious chronic respiratory disorder. It is characterized by various clinical symptoms ranging from high-grade fever with headache, lethargy, and thrombocytopenia to rapid pulmonary edema and increased vascular permeability. The vascular fluid leakage results in noncardiogenic edema followed by complete respiratory dysfunction, multiorgan failure, cardiogenic shock, and death. Various pathogenesis has been suggested, including immune-mediated cell injury, cytokine storm, and high VEGF responses from intercellular junctions resulting from the virus–integrin interactions (4). Since the majority of deaths are caused by cardiac failure and hypoperfusion rather than hypoxia, several researchers prefer the term “hantavirus cardiopulmonary syndrome” rather than hantavirus pulmonary syndrome (5). No specific drug has been discovered to treat Sin Nombre infection; however, ribavirin, a broad-spectrum antiviral agent, is mainly used for supportive therapy for the treatment of HCPS, but no effective results are reported yet. Hence, designing a vaccine against HCPS is the challenge to global health to tackle Sin Nombre orthohantavirus infection. There is no effective and FDA-approved vaccine against SNV; however, many attempts have been made to develop effective vaccines, including conventional, molecular approaches, subunit, and plasmid DNA delivery by gene gun. All these approaches end without any positive progress. Multi-epitope–based vaccines have precedence over traditional vaccinology because of their highly distinctive features, including safety, population coverage, antigenicity, immunogenicity, conservancy, cheapness, and being less arduous (6).

A safe and effective vaccine was discovered by Sabin and Salk against poliovirus using Pasteur’s vaccinology approach. Pasteur’s vaccinology role led to the formulation of the BCG vaccine against Mycobacterium tuberculosis. Nonetheless, such a vaccinology technique fails for numerous bacterial diseases that cannot be cultivated on culture media. Moreover, culture-based vaccines reveal antigenic unevenness mainly in the case of Neisseria meningitides. Currently, bioinformatics and reverse vaccinology approaches are used to predict highly conserved as well as immunodominant peptide epitopes from the epitopes databases (7). This provides a platform for the development of multi-antigenic epitope-based vaccines against several bacterial pathogens. The modern research in immunology and vaccinology is excellently contributed to by immunoinformatics. Several studies worked on designing extracellular vesicle-based vaccines that have higher biosafety and efficiency in triggering an immune response. In general, a good vaccine candidate must have properties to trigger a specific B cell cytotoxic T cell response in the host body (8). Several databases collect experimentally verified vaccines and vaccine components, while *in silico* tools provide computational methods to predict and design new vaccines and vaccine components (8). This study was designed to develop a genome based vaccine against SNV using several reverse vaccinology, bioinformatics, immmunoinformatics, and biophysical approaches.

## Research methodology

**Figure 1:**
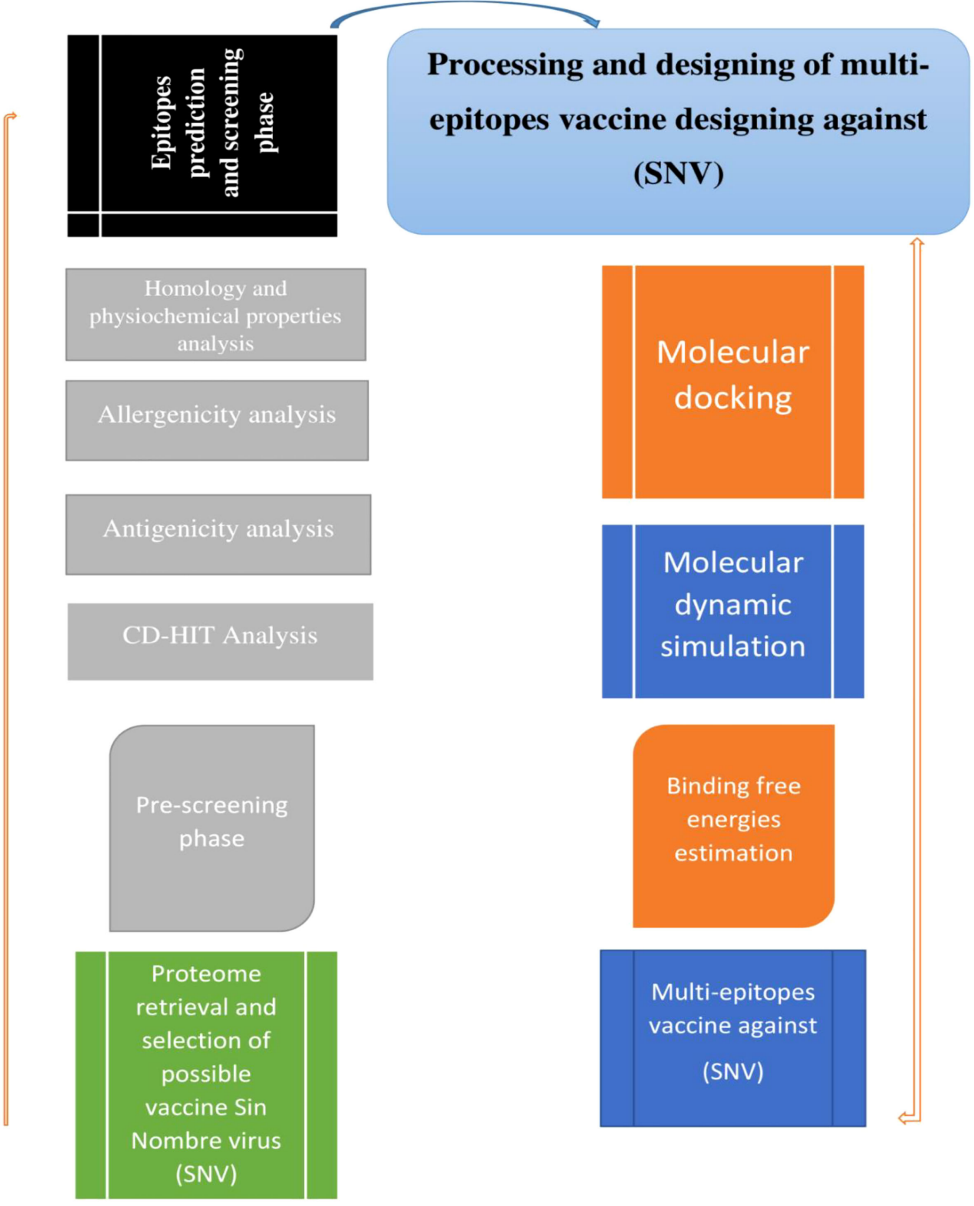
Schematic representation of research methodology.

**Figure 1** depicts the overall study flow that was utilized to build a multi-epitope peptide vaccine to combat the Sin Nombre orthohantavirus.

### Proteome retrieval and selection of possible vaccine candidate

The study began with the retrieval of a complete proteome of SNV from the national center of biotechnology information (9). After proteome retrieval, redundant proteins were discarded using an online cluster database at high identity with tolerance (CD-HIT) suite server, and the nonredundant proteins were subjected to further analysis (10). Antigenicity analysis was performed to analyze antigenic proteins through the Vaxijen 2.0 (11) webserver at a 0.4 threshold. Allergenicity analysis was performed *via* the Allertop 2.0 server to remove the allergic protein sequence that can cause an allergic immune response (12). Homology was checked through BLASTp against humans (taxid: 9606) and the protein sequence showing similarity with humans was removed (9). Physiochemical property analysis was performed through an online web server, ProtParam (13), and all of the unstable proteins were discarded. Proteins having an instability index >40 were considered unstable and removed while the remaining probable antigenic, human nonsimilar, nonallergic, and stable proteins were subjected to epitope mapping.

### Epitope prediction and screening phase

The shortlisted proteins were used for B and T cell epitope prediction using Bepipred Linear Epitope Prediction 2.0 in the IEDB online webserver for B cell epitopes (14). For T cell epitopes, T-Cell Epitope Prediction resources were used in IEDB (15). The epitopes were further screened for antigenicity, allergenicity, toxicity, and water solubility using vaxijen 2.0 http://www.ddg-pharmfac.net/vaxijen/VaxiJen/VaxiJen.html, allertop 2.0 https://www.ddg-pharmfac.net/AllerTOP/, ToxinPred http://crdd.osdd.net/raghava/toxinpred/, and the peptide solubility calculator http://crdd.osdd.net/raghava/toxinpred/, respectively (16). The screened epitopes were used in a multi-epitope-based vaccine against SNV (17).

### Processing and designing a multi-epitope vaccine against SNV

The shortlisted epitopes were used in multi-epitope vaccine design. The epitopes were connected through AAY linkers. Additionally, the construct was also linked with cholera-toxin b subunit adjuvant *via* an EAAAK linker (18). Physiochemical properties were assessed through the ProtParam tool (19). Population coverage analysis was done through the IEDB online database http://tools.iedb.org/population/download/. The 3-D structure of the vaccine was predicted, and disulfide engineering was done through Designed.20 webserver (20), where all the enzymatic sensitive residues were replaced by cysteine amino acid residues (21). The secondary structure was generated by pdbsum generate https://bio.tools/pdbsum_generate, and the Ramachandran plot was analyzed (22).

### Molecular docking study

Molecular docking analysis was performed using the online Patch Dock Server (23). In docking analysis, the binding affinity of vaccines with TLR-4 and TLR-8 were analyzed. The server generated 20 docked complexes in each case. The docked complexes were further refined through the FireDock webserver, and 10 docking complexes were refined (24). On the basis of the lowest binding energy, in the case of vaccines with TLR4 and TLR8, the top complexes were subjected to simulation (25).

### Molecular dynamic simulation and MMGB/PBSA analysis

Molecular dynamic (MD) simulation of top-docked complexes was performed using AMBER software at 60 ns (26). MD simulation was done to check the binding mode and dynamic behavior of the docked molecules. An antechamber program was used for generating docked complexes, and the complexes were solvated in a (TIP3P) sol box (27). For intermolecular interaction (Ff14SB), a force field was used (28). For neutralization of TLR-4 and TLR-8, 9, 8, and 7, sodium counterions were added (21). After the system preparation phase, preprocessing was done in which the system was ready for the production phase. By steepest decent and conjugate base algorithms, the energy was maintained, and the system was heated to 300K. For constraint of hydrogen bonding, the SHAKE algorithm was utilized (29). Through the Berendsen algorithm, trajectories of the simulation were used, and the trajectories were analyzed through PPTRAJ (30). In MMGBSA (31) and MM/PBSA (32) analysis, the net binding free energies for vaccines TLR-4 and TLR-8 were calculated. A total number of 100 frames were determined between the solvated and gas phases (33).

## Results

### Subtractive proteomics pipelines

**Figure 2:**
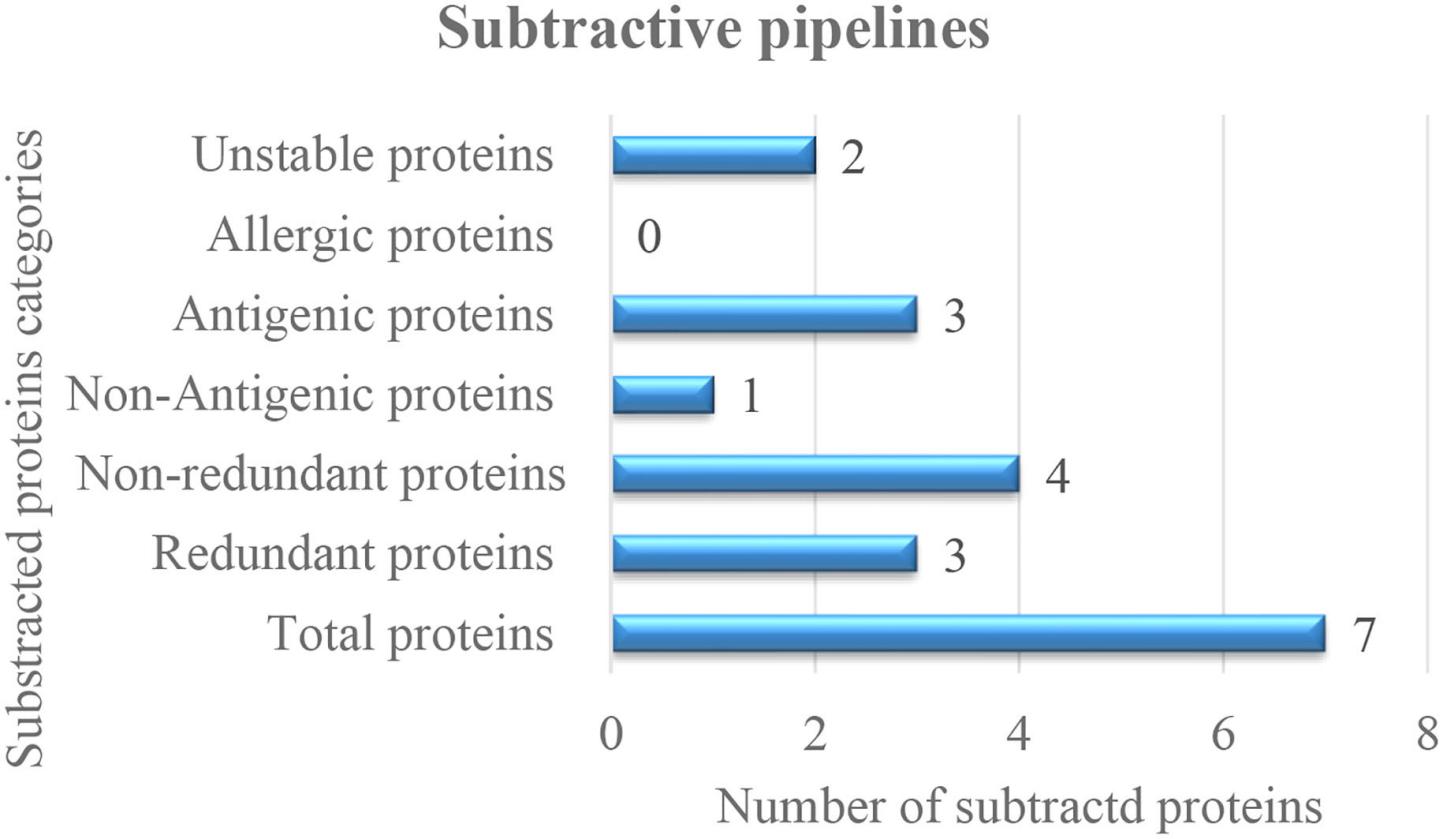
Shortlisted proteins for good vaccine candidate 3.2 Mapping of epitopes.

The study commenced with the retrieval of the complete genome of SNV two strains (accessions GCA_002830985.1 and GCA_000854765.1_) *via* GenBank assembly. After genome retrieval, conserved protein sequences were determined through CD-HIT analysis in which the redundant proteins were discarded and the nonredundant proteins were selected. Among the total of seven proteins, three proteins were predicted as redundant protein sequences, and four were predicted as nonredundant proteins. In four nonredundant proteins, one protein was predicted to be nonantigenic and three were predicted to be probably antigenic with a 0.4 threshold value. In total, there were three antigenic proteins; one protein was predicted as stable, and two proteins were predicted unstable. Finally, only one protein was selected for epitope selection. The subtracted protein categories and numbers are presented in the following **Figure 2**.

### Mapping of epitopes

The final shortlisted protein glycoprotein precursor (GPC) of surface glycoproteins (accession number >AAC42202.1) was used for epitope prediction. The predicted epitopes are tabulated in the following **Table 1**.

**Table 1:** Predicted B cell peptide epitopes and T cell epitopes with the least percentile score (P.S).

Selected proteins	B-CELLS- EPITOPES	MHC-II	P.S	MHC-I	P.S
>AAC42202.1 Glycoprotein precursor (GPC) of surface glycoproteins	IESSCNFDLHVPATTTQKYNQVDWTKKSSTTESTNAGATTFEAK	TESTNAGATTFEAK	0.68	STNAGATTF	0.12
	VLNPRGEDHDPDQNGQG	VPATTTQKYNQVDWTKKSST	15	VPATTTQKY	0.01
		IESSCNFDLHVPAT	8.5	QVDWTKKSST	15
				IESSCNFDL	0.46
		LNPRGEDHDPDQNG	60	NFDLHVPAT	16
				GEDHDPDQNG	4.8
	DCTEEGLAASPPHLDRVTGYNQIDSDKVYDDGAPP	TGYNQIDSDKVYDD	6	YNQIDSDKVY	0.03
		DCTEEGLAASPPHLDRV	15	GLAASPPHL	0.05
				DCTEEGLAA	11
	QGNTVSGFQRMMATRDSFQSFNVTEPHITSNRLEWIDPDSSIK	SGFQRMMATRDSF	0.09	RMMATRDSF	0.07
				SGFQRMMATR	0.63
		FQSFNVTEPHITSN	7.1	QSFNVTEPH	1.9
		TSNRLEWIDPDSSIK	4.3	LEWIDPDSSI	0.45
				TSNRLEWID	11
	CVFGDPGDIMSTTSGMRCPEHTGSFRKICGF	PGDIMSTTSGMRCP	6.4	DIMSTTSGMR	0.33
		RCPEHTGSFRKICGF	44	GSFRKICGF	0.38
				RCPEHTGSF	1.5
	KKYAYPWQTAKCFFEKDYQYETSWGCNPPDCPGVGT	AKCFFEKDYQYETS	4.9	KCFFEKDYQY	0.7
		KKYAYPWQTAKCFF	26	YPWQTAKCF	0.02
		TSWGCNPPDCPGVGT	55	GCNPPDCPGV	12
	DFALASSSSYSYRRKLVNPANQEET	DFALASSSSYSYRRKL	2.3	FALASSSSY	0.01
				SSYSYRRKL	0.98
		DFALASSSSYSYRRKLV	2.6	FALASSSSY	0.1
				SSYSYRRKLV	2.8
	ADTPLMESGWSDTAHG	TPLMESGWSDTAHG	15	MESGWSDTA	14
	KSLKRPEVRKGCY	KSLKRPEVRKGC	27	SLKRPEVRK	0.03
	TAKVPSTETTETMQGI	VPSTETTETMQGI	29	ETTETMQGI	0.02

### Epitope prioritization phase

In the epitope prioritization phase, the predicted epitopes were further checked for antigenicity, allergic response, water solubility, and toxicity. Afterward, only antigenic, nontoxic, nonallergic and water soluble epitopes were shortlisted for vaccine model construction. The shortlisted epitopes are mentioned in the following **Table 2**.

**Table 2:** Selected epitopes for vaccine construct modeling.

S.No	Shortlisted epitopes	Percentile score	Antigenicity	Allergenicity	Water solubility and toxicity
1	QVDWTKKSST	15	1.3826	Nonallergic	Good water solubility and nontoxic
2	GLAASPPHL	0.05	0.7461		
3	SSYSYRRKLV	2.8	0.5629		
5	MESGWSDTA	14	0.7445		

### Multi-epitopes vaccine design phase

**Figure 3:**
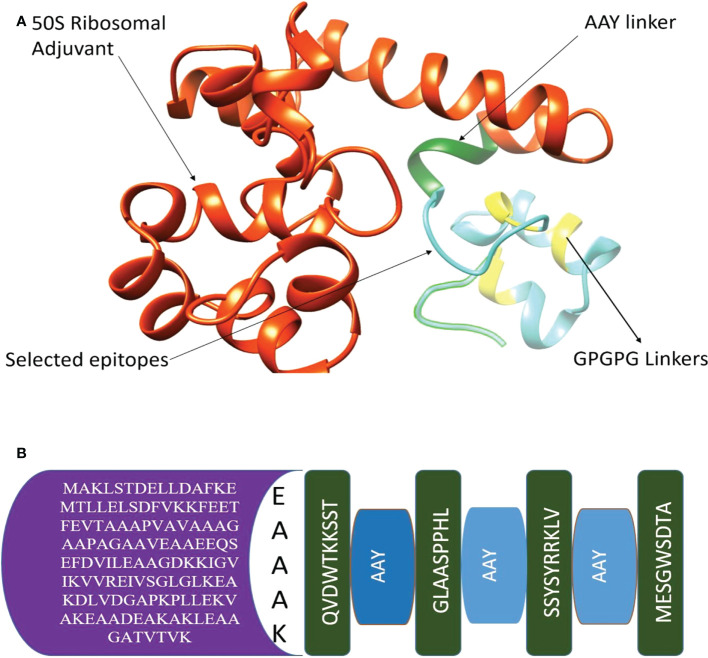
**(A)** 3-D structure of chimeric vaccine **(B)** Adjuvant, EAAAK, AAY linkers, and selected epitopes.

Multi-epitope vaccines consist of different epitopes with various lengths. The filtered shortlisted epitopes were linked by GPGPG and AAY linkers, and additionally, the construction was connected to 50S ribosomal adjuvant. The 3-D structure is presented in the following **Figure 3A**, and the schematic presentation is given in **Figure 3B**.

**Figure 4:**
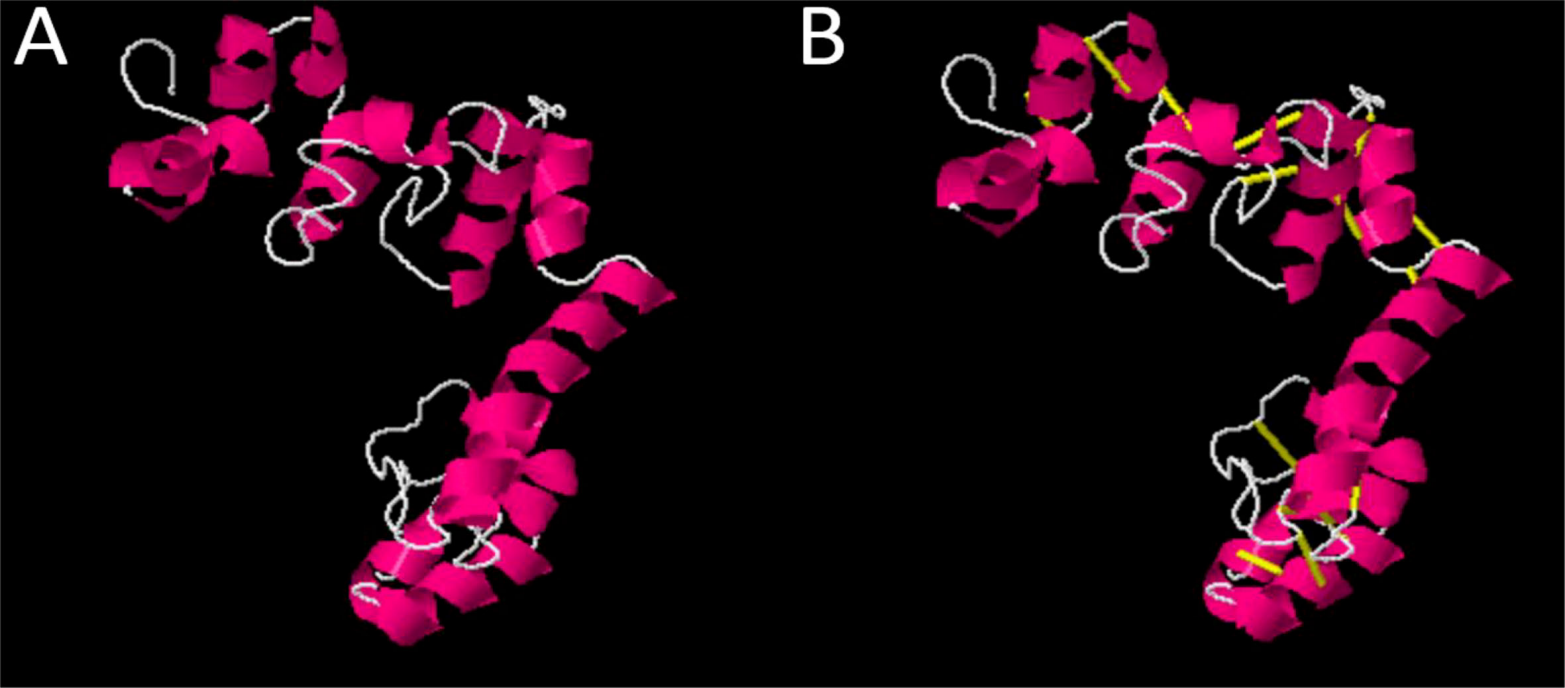
**(A)** Original vaccine structure **(B)** Mutated vaccine structure; in the mutated structure, yellow lines represent mutated residues.

In the next step, physiochemical properties were analyzed. The vaccine construct comprises 182 amino acids with a molecular weight of 19.03770. Antigenicity was calculated, and the vaccine construct was predicted as antigenic with a 0.4787 score. The instability index was calculated as 26.52, which classifies the protein as probably stable. Furthermore, to improve structural stability, the vaccine structure was modified, and overall, the enzymatic sensitive residues were replaced by cysteine amino acid residues in disulfide engineering processes. The Disulfide by Design 2.13 tool was used to analyze the vaccine sequence, and a total of 10 potential residue pairings, LEU21-PHE24, Chi3106.79, Energy 4.97, VAL34-ALA37 Chi3 101.1, Energy 1.45, ASP64-ILE84 Chi3 -99.48, Energy 2.39, ALA69-VAL80 Chi 84.61, Energy 5.42, ARG82-SER86 Chi3 -62.06, Energy 7.53, TRP139-LEU150 Chi3 126.68, Energy 5.97, THR145-ALA157 Chi3 88.39, Energy 5.97, HIS156-TYR173 Chi3 -76.58, Energy 3.37, SER161-ARG166 Chi3 105.31, Energy 1.57, MET174-THR181 Chi3126.83, Energy 2.19, that could form a disulphide bond were discovered. The original and mutated structure of the vaccine is presented in **Figure 4A, B**.

### Population coverage by epitopes

**Figure 5:**
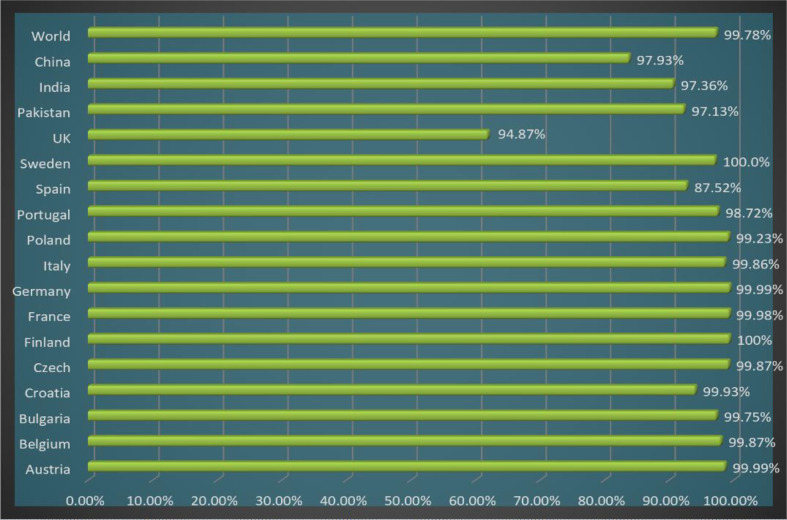
Chimeric vaccine population coverage analysis for the world and different countries.

In the population coverage analysis, we observed that the shortlisted epitopes for vaccine candidates unevenly correspond to 99.78% of the world’s population while other countries and their population coverage percentages are mentioned in **Figure 5**.

### Secondary structure analysis

**Figure 6:**
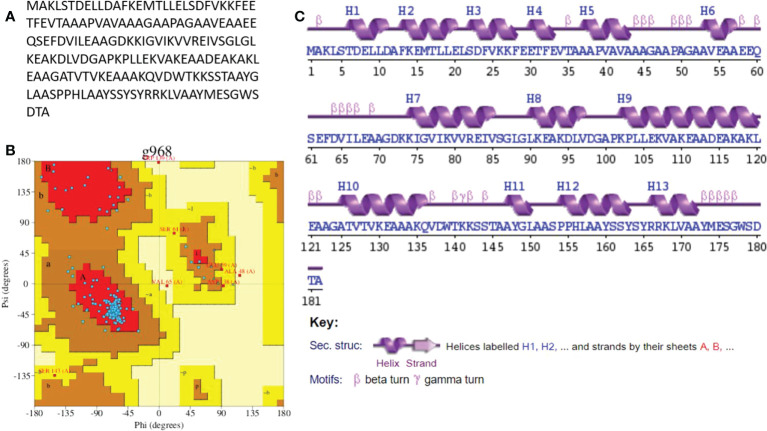
**(A)** Vaccine construct sequence **(B)** Ramachandran plot **(C)** secondary structure.

The secondary structure of the model’s vaccine was predicted. The structure contains 102 (56.0%) alpha helix, allowing for Ramachandran plot analysis, which was carried out through PROCHECK. The refined model has 137 (83.5%) residues in the most favored regions and 20 (12.2%) residues in additionally allowed regions. However, four (2.4%) residues were observed in the generously allowed region while there were three in the disallowed regions (1.8%). A complete explanation of the improved vaccine secondary structure is presented in **Figure 6**.

### Molecular docking and refinement analysis

**Figure 7:**
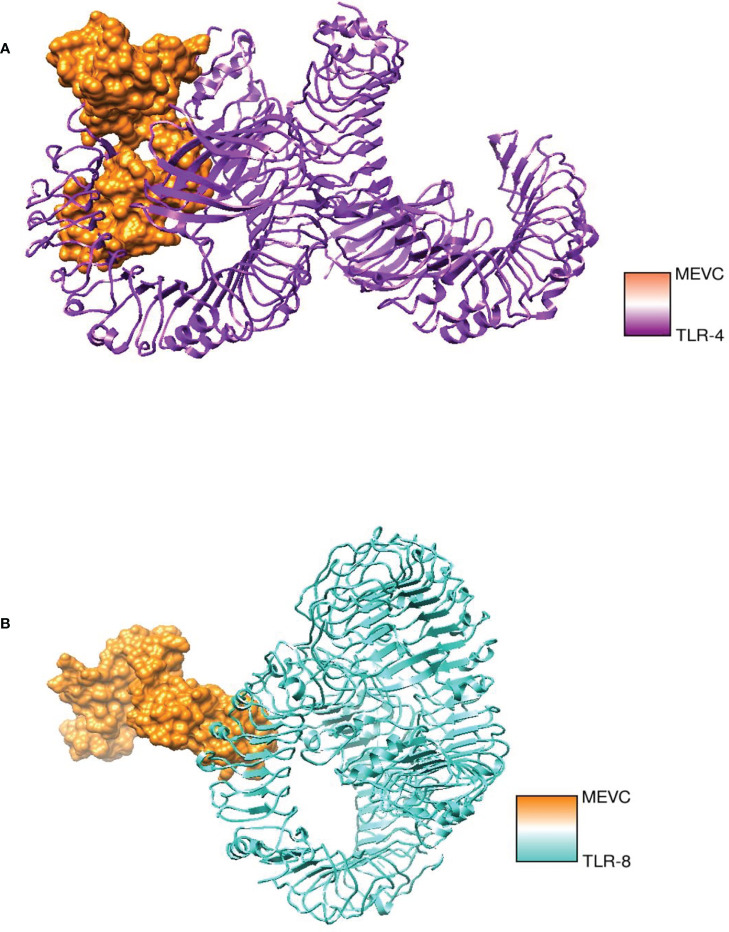
**(A)** Chimeric vaccine with TLR-4 **(B)** Chimeric vaccine with TLR-8.

Molecular docking analysis was performed with human immune cell receptors TLR-4 and TLR-8 using an online patch docked web server. The server generated 20 docked solutions (**Table S 1-2**), and the docking results were further refined using the Fire Dock server. The solution number, global energy attractive VdW and repulsive VdW, atomic center energy (ACE), and hydrogen bonds (HB) of the top 10 docked complexes are presented in the following **Tables 3**, **4**. The docked confirmation is presented in **Figure 7A, B**.

**Table 3:** Docking score of refined complexes of TLR-4 and vaccine.

Rank	Solution number	Global energy	Attractive VdW	Repulsive VdW	ACE	HB
1	10	-12.52	-16.17	11.11	5.91	-2.10
2	3	-1.47	-31.45	30.64	10.13	-2.04
3	7	-0.79	-42.37	25.16	15.04	-3.53
4	2	2.87	-1.56	0.00	0.92	0.00
5	9	11.79	-22.40	5.90	12.70	-1.09
6	6	11.94	-37.68	19.40	17.68	-4.31
7	5	20.09	-8.11	6.70	5.57	-2.82
8	1	1875.66	-72.30	2472.89	5.24	-5.91
9	8	2292.19	-53.59	2955.83	4.17	-5.40
10	4	2964.90	-54.48	3714.76	17.84	-3.86

Docked complexes having the lowest binding energy score, atomic center energy (ACE), and hydrogen bonding (HB) were considered the best docked solution.

**Table 4:** Docking score of refine complexes of TLR-8 and vaccine.

Rank	Solution number	Global energy	Attractive VdW	Repulsive VdW	ACE	HB
1	5	-5.42	-18.65	6.94	17.72	-3.28
2	4	22.47	-4.33	0.00	2.84	0.00
3	9	105.47	-7.65	106.17	7.05	-1.44
4	10	196.42	-27.98	259.70	7.89	-3.63
5	3	766.83	-71.11	1006.64	18.26	-6.62
6	7	2057.46	-77.56	2648.52	13.41	-2.57
7	8	2253.83	-47.09	2839.81	13.41	-3.12
8	6	5339.28	-115.09	6782.90	23.30	-7.55
9	1	7192.97	-116.24	9147.96	12.78	-10.18
10	2	8857.61	-184.86	11324.62	45.98	-24.12

Docked complexes with lowest binding energy score, atomic center energy (ACE), and hydrogen bonding (HB) were considered the best docked solution.

### Normal mode analysis of the vaccine TLR-4 and TLR-8

**Figure 8:**
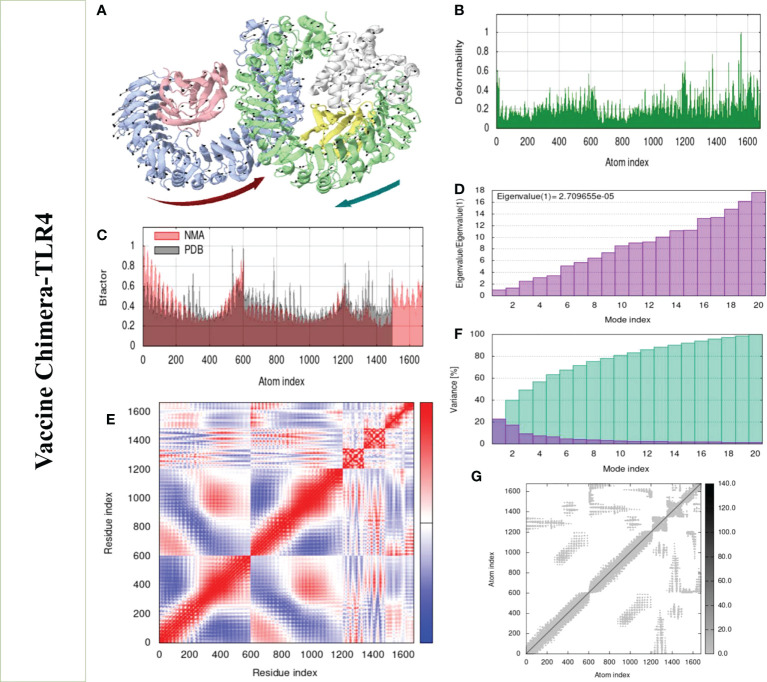
Normal mode analysis of the vaccine-TLR-4. **(A)** MOBILITY of refined protein-protein complex. **(B)** In relation to atom deformability. **(C)** In relation to atom B factors. **(D)** Eigenvalue in relation to modes. **(E)** In relation to residue covariance. **(F)** Variance of structural changes. **(G)** In relation to atom elastic networks.

**Figure 9:**
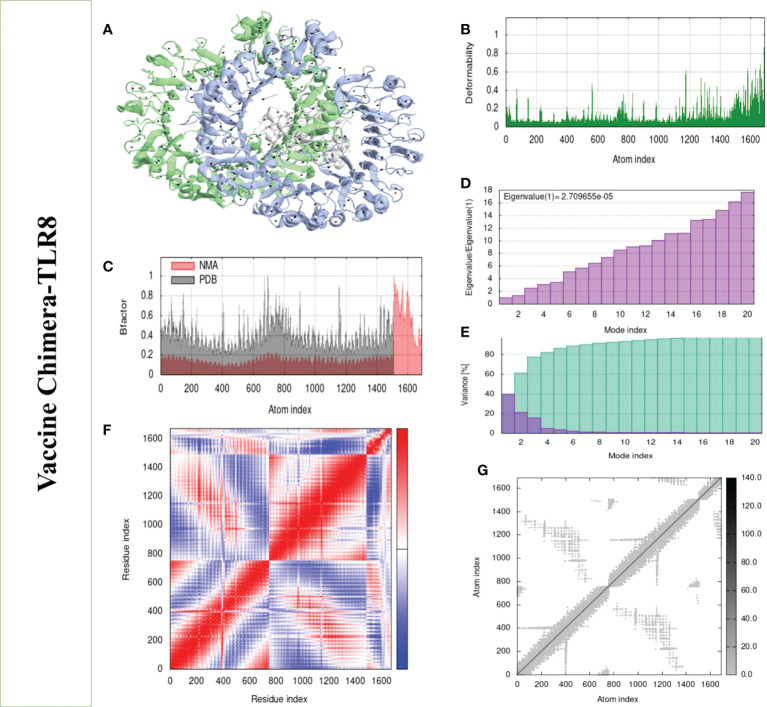
Normal mode analysis of the vaccine-TLR-8. **(A)** Mobility of refined protein–protein complex. **(B)** In relation to atom deformability. **(C)** In relation to atom B factors. **(D)** Eigenvalue in relation to modes. **(E)** The variance of structural changes. **(F)** In relation to residue covariance. **(G)** In relation to atom elastic network.

Structural analysis of top-hit docked complexes was evaluated through the iMod online web server. The server adjusts the complex’s force field concerning various time intervals for docked structure analysis. The arrow in **Figures 8**, **9A** represents the direction of residues while the line represents the movement in the 3-D model. Here, we observed that the TLR-4 and TLR-8 receptors and vaccine allow their chain to interact with each other’s. The generated docked complexes vaccine with TLR-4 and TLR-8 reveal reduced alteration at the point of each residue’s capability level as mentioned in **Figures 8**, **9B**. The following beta factor values align and support the RMSD results shown in **Figure 8C** for the vaccine with TLR-4 and **Figure 9C** for the vaccine with TLR-8. The eigenvalues for the TLR-4 docked cluster (**Figure 8D**) and TLR-8 docked cluster (**Figure 9D**) are 2.709655e – 05 and 2.856198e – 6, respectively. Maximal interaction with interface protein residues was reported in all heat maps with low RMSD and maximum co-relation sites, i.e., common eigenvalue and normal mode variance, as seen in inverse co-relation (**Figures 8E**, **9E**). Additionally, the covariance matrix provides a visual representation of coupling between residual pairs. **Figures 8F**, **9F** show that this coupling may be due to correlated, uncorrelated, or anticorrelated motions. An elastic network model was produced using NMA at the end (**Figures 8**, **9G**).

### Molecular dynamic simulation analysis

**Figure 10:**
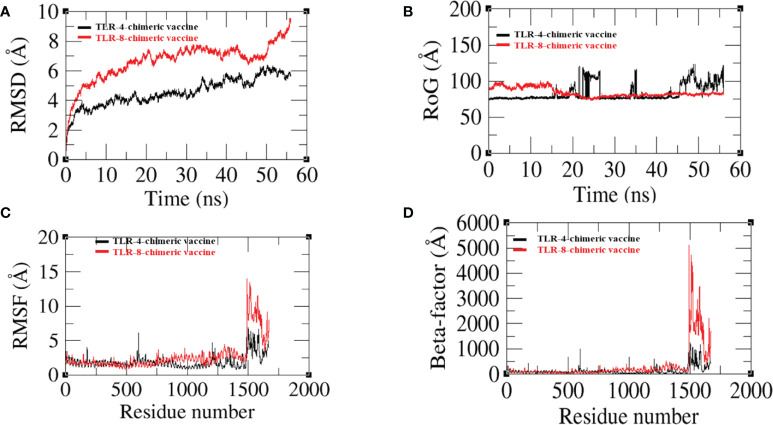
**(A)** Root mean square deviation (RMSD) **(B)** Radius of gyration (RoG) **(C)** Root mean square fluctuation (RMSF) **(D)** Beta-factor.

The dynamic movement of the chimeric vaccine with TLR-4 and TLR-8 was observed in MD simulation analysis. RMSD, RMSF, RoG, and Beta factors of top docked complexes were analyzed. In RMSD, we observed that the binding ability of the vaccine to TLR-4 is more compared with TLR-8, where the graph deviated more at 8.9 (Å) at 55 ns as presented in **Figure 10A**. Similarly, the residual level fluctuation of the vaccine with immune receptors was evaluated using RMSF, and we observed that the graph of vaccine-TLR-8 fluctuates more than the graph of vaccine-TLR-4, but in the end, both graphs demonstrate stability as shown in **Figure 10C**. Next, a radius of gyration analysis was performed to analyze the docked complexes’ compactness and relaxation. As shown in **Figure 10B**, the graph demonstrates stability with low variation, indicating adequate compactness in the docked structure as evaluated by RoG analysis. Moreover, the beta factor of the docked complex validates the RMSD as presented in **Figure 10D**. Overall, the simulation results reveal that the vaccine has a good binding affinity with immune cell receptors.

### Free binding energies estimation

Binding free energies were calculated through MMGBSA and MMPBSA analysis. The MMGBSA analysis calculated -122.17 and -125.4 net binding energies for the TLR-8- and TLR4-vaccine complex, respectively, while the MMPBSA analysis estimated -115.63 and -118.19 for the TLR-8- and TLR4-vaccine complex, respectively, as mentioned in **Table 5**.

**Table 5:** Binding free energies calculation values and parameters.

Energy parameter	TLR-8-Vaccine complex	TLR4-Vaccine complex
MM-GBSA		
**VDWAALS**	-68.62	-6.55
**EEL**	-85.14	-81.32
**Delta G gas**	-149.90	-150.87
**Delta G solv**	27.60	25.47
**Delta Total**	-122.20	-125.4
MM-PBSA		
**VDWAALS**	-67.62	-69.55
**EEL**	-85.14	-81.32
**Delta G gas**	-149.90	-150.87
**Delta G solv**	34.55	32.68
**Delta Total**	-115.90	-118.19

## Discussion

SNV is the infectious agent of acute viral pneumonia in humans, HVPS, with a 50% death rate (34). Hantaviruses are a major public health concern due to the emergence of novel strains, and the lack of proper strategies can quickly affect global health. The vaccine is an alternative and possible way to eradicate infectious diseases (35). With several limitations in Pasteur vaccinology like having a high-risk factor pathogen or being time-consuming, highly expensive, and many more, the advancement in reverse vaccinology, bioinformatics, and immunoinformatics approaches have made it possible to predict such good prophylactics and therapeutics agents like drug and vaccine candidates with suitable accuracy, time saving, and cost-effectiveness (36). Hence, in this study, the aforementioned approaches were utilized for the design of a vaccine candidate against SNV. In the study, a total of seven core proteins were utilized (37). Since duplicate representations of redundant proteins typically occur in the same strains, three redundant proteins from the total core proteins were removed, and four were processed further (38). Antigenic proteins induced immune responses in the side host body against particular pathogens (39), so only antigenic proteins were shortlisted, and the nonantigenic proteins were discarded (40). Allergic response in vaccination is a risk factor for the host. To avoid this risk, all the shortlisted proteins were analyzed for a homology check, and we removed all those sequences having similarities with human and human normal microbiota. The purpose of homology analysis was to reduced unwanted autoimmune reactions and protect microbiota of the host body (41). Next, the probable antigenic shortlisted proteins were subjected to epitope prediction. Multi-epitope vaccines were constructed mainly of epitopes having the ability to induce a proper immune response in the host body against the particular pathogen (42). Previously, researchers designed vaccines against hantavirus (18), and their proposed vaccine showed an immune system–inducing capability against hantavirus. After epitope prediction and prioritization, the vaccine construct was designed. Furthermore, the 3-D structure was predicted (43), and for the retention of structure stability, disulfide bonds were incorporated (20). A molecular docking study was carried out for the interaction of the vaccine with immunological mediator cell receptors to activate the immune system. Furthermore, similar approaches were applied to develop a vaccine against several bacterial species using the same methodology (18). Molecular dynamic simulation was used to analyze the dynamic behavior, and the results showed that the vaccine and immune cell receptors have a stable binding ability. The proposed model vaccine against SNV could essentially aid in eliciting an immune response; however, as the work is computationally based, experimental validation is required to further validate the results since our all *in silico* based results map all key criteria for potential vaccine chimera, and the model vaccine candidate can be broadly used against SNV. However the study has several experimental limitations, the main objective being that the work is to provide a theoretically based vaccine construct for experimentalists to check the designed vaccine’s immune protective efficacy against SNV *in vivo*; in experimental study, the paper findings will definitely speed up vaccine development against said pathogen.

## Conclusion

SNV has been identified as the primary agent of HCPS, and the lack of an adequate vaccine against this disease is a major public health concern. The vaccine is an alternative way to tackle bacterial and viral species although the formulation of vaccines utilizing the Pasteur vaccinology approach has certain challenges, including allergenicity, pathogen severe immunogenicity, and toxicity. To tackle this issue, reverse vaccinology, bioinformatics, and immunological informatics approaches are crucial for the development of vaccine in the current era. A multi-epitope-based vaccine was designed in this manuscript, and the vaccine construct demonstrated its effectiveness for generating a healthy immune response in the host against the targeted pathogen. However, as the whole study is based on a computational approach, further experimental validation is recommended to ensure the results.

## Data availability statement

The datasets presented in this study can be found in online repositories. The names of the repository/repositories and accession number(s) can be found in the article/**Supplementary Material**.

## Author contributions

The author contributed to conception and design of the study; analysis and interpretation of data; drafting, revising and approval of the submitted version.

## Acknowledgments

The author acknowledges Prince Sattam Bin Abdulaziz University and the Deanship of Scientific Research at Prince Sattam Bin Abdulaziz University for their support and for providing the necessary tools to conduct this study. The author thank Dr. Safar M. Alqahtain (College of Pharmacy, Prince Sattam Bin Abdulaziz University, Al Kharj, Saudi Arabia) and Dr. Maaged A. Akiel (Department of Clinical Laboratory Sciences, College of Applied Medical Sciences, King Saud bin Abdulaziz University for Health Sciences (KSAU-HS), King Abdullah International Medical Research Center (KAIMRC), Riyadh, Saudi Arabia) for their expertise and assistance throughout all aspects of our study and for their help in writing the manuscript.

## Conflict of interest

The author declares that the research was conducted in the absence of any commercial or financial relationships that could be construed as a potential conflict of interest.

## Publisher’s note

All claims expressed in this article are solely those of the authors and do not necessarily represent those of their affiliated organizations, or those of the publisher, the editors and the reviewers. Any product that may be evaluated in this article, or claim that may be made by its manufacturer, is not guaranteed or endorsed by the publisher.
