# Supplementary material for: Integrativesubtractive proteomics, immunoinformatics, docking, and simulation approaches reveal candidate vaccine against Sin Nombre orthohantavirus
Source: Front Immunol. 2022 Nov 11;13:1022159. doi: 10.3389/fimmu.2022.1022159 (PMC9692015; doi:10.3389/fimmu.2022.1022159)
Supplement: Supplementary file 1 [file Table_1.docx]

**Table S1.** Docking score of TLR-4 and Vaccine

| **Solution No** | **Score** | **Area** | **ACE** | **Transformation** |
| --- | --- | --- | --- | --- |
| 1 | 20698 | 2825.40 | 302.47 | 0.27 0.13 -0.42 -60.59 23.16 -9.23 |
| 2 | 20636 | 3537.60 | 343.89 | -2.38 0.11 -2.46 18.71 16.92 -65.39 |
| 3 | 18128 | 2882.20 | 385.88 | -2.94 1.04 0.41 -17.92 -15.94 -7.20 |
| 4 | 17488 | 3095.00 | 169.10 | -2.21 0.10 -2.76 22.20 13.93 -67.94 |
| 5 | 17164 | 2541.30 | 140.64 | -3.11 -0.91 1.60 50.20 6.11 -59.42 |
| 6 | 17028 | 2873.00 | 444.33 | 0.68 0.79 1.67 -55.23 -5.16 9.59 |
| 7 | 16876 | 2597.30 | 469.23 | 2.00 -1.30 2.40 -41.43 15.74 16.07 |
| 8 | 16534 | 2546.90 | 484.26 | 2.69 0.43 3.04 -42.37 54.58 -30.64 |
| 9 | 16478 | 3090.50 | 318.43 | 1.30 0.90 0.85 -53.34 -8.36 8.69 |
| 10 | 16218 | 2287.10 | 202.23 | 1.85 -0.77 1.81 -29.42 27.29 -17.21 |
| 11 | 16202 | 2749.60 | 353.07 | -0.89 -0.79 -3.14 20.78 -21.79 -61.33 |
| 12 | 16130 | 2412.50 | 120.43 | 2.72 -0.19 2.06 3.83 47.97 -25.37 |
| 13 | 16020 | 1917.90 | 474.33 | -2.69 -0.39 2.52 -33.36 22.09 -27.15 |
| 14 | 15898 | 2729.00 | 81.42 | -2.30 -0.29 -1.46 -53.13 28.46 -64.40 |
| 15 | 15894 | 2768.00 | 291.41 | 0.98 -0.08 -0.41 7.11 22.27 -9.62 |
| 16 | 15852 | 2342.60 | 187.35 | -0.23 1.32 -0.17 -49.03 60.42 -20.69 |
| 17 | 15822 | 1999.90 | 371.37 | 0.75 0.74 -2.59 -37.25 28.72 -19.83 |
| 18 | 15756 | 2606.30 | 165.62 | 1.83 -1.02 1.60 -27.66 33.48 -14.89 |
| 19 | 15722 | 2091.30 | 487.52 | 2.24 -0.11 -2.64 -66.57 54.55 -5.46 |
| 20 | 15658 | 2234.10 | 383.97 | -2.43 -0.26 -1.58 -0.70 -26.99 -52.93 |

**Table S2.** Docking score of TLR-8 and Vaccine

| **Solution No** | **Score** | **Area** | **ACE** | **Transformation** |
| --- | --- | --- | --- | --- |
| 1 | 21220 | 3934.20 | 329.70 | 1.73 0.72 2.02 75.19 -7.61 71.58 |
| 2 | 18918 | 4649.40 | 396.23 | -2.13 0.16 -3.01 46.35 -11.69 30.24 |
| 3 | 18780 | 3478.80 | 399.18 | -1.28 -0.97 -1.98 70.42 -15.11 -3.52 |
| 4 | 18748 | 2918.10 | 235.16 | -1.27 -0.77 -2.04 70.20 -20.67 -5.61 |
| 5 | 18624 | 2899.90 | 390.67 | 2.57 -0.41 3.02 46.97 34.38 72.47 |
| 6 | 18208 | 4104.20 | 418.29 | -1.48 -0.29 -1.72 61.30 -10.07 -7.62 |
| 7 | 17796 | 2805.30 | 463.97 | 1.26 0.41 0.48 29.26 -8.90 94.34 |
| 8 | 17668 | 3056.70 | 344.54 | 0.40 -1.39 -0.04 37.66 -20.80 8.05 |
| 9 | 17630 | 2504.20 | 467.15 | 0.29 -0.87 2.98 35.01 -8.96 89.79 |
| 10 | 17626 | 3642.80 | 107.18 | 2.87 0.02 -2.20 10.10 28.82 55.30 |
| 11 | 17412 | 3676.40 | 47.76 | -2.27 0.50 -3.07 41.46 -8.19 32.90 |
| 12 | 17286 | 3610.60 | 31.50 | 1.46 0.48 -2.71 53.23 20.39 81.18 |
| 13 | 17134 | 2133.20 | 400.54 | 0.58 -0.37 0.06 31.86 -25.18 20.61 |
| 14 | 17106 | 4171.30 | 402.14 | -2.47 0.06 0.93 30.54 -1.06 38.19 |
| 15 | 16970 | 3199.60 | 297.76 | 2.39 -0.44 0.41 66.00 -32.76 69.79 |
| 16 | 16912 | 2553.00 | 326.74 | 1.51 0.33 -2.40 -3.36 -9.74 46.25 |
| 17 | 16760 | 4296.10 | 198.08 | -2.17 0.34 3.02 42.40 -11.31 30.68 |
| 18 | 16760 | 2305.50 | 437.71 | -0.59 -0.10 2.22 1.32 -54.18 -0.08 |
| 19 | 16710 | 2519.90 | 231.06 | -0.10 0.29 -1.95 46.15 -29.10 9.71 |
| 20 | 16636 | 3364.80 | 464.32 | -1.20 -0.89 2.63 35.61 -66.93 -1.84 |
